# Healthcare provider challenges to early detection of cervical cancer at primary healthcare level in Rwanda

**DOI:** 10.1016/j.gore.2021.100810

**Published:** 2021-06-17

**Authors:** Charles Nkurunziza, Rahel Ghebre, Urania Magriples, Diomede Ntasumbumuyange, Lisa Bazzett-Matabele

**Affiliations:** a Department of Obstetrics and Gynecology, College of Medicine and Health Sciences, University of Rwanda, P.O. Box 5978, Kigali, Rwanda; b Department of Obstetrics and Gynecology, University of Minnesota Medical School, 395 MMC, 420 Delaware Street S.E., Minneapolis, MN 55455, USA; c Department of Obstetrics and Gynecology, Yale University School of Medicine, 333 Cedar St., New Haven, CT 06510, USA; d Department of Obstetrics and Gynecology, University of Botswana, Private Bag 00713, Gaborone, Botswana

**Keywords:** Global health, Cervical cancer, Early detection, Primary healthcare, Rwanda, DNA, Deoxyribonucleic acid, HC, Health Center, HPV, Human papilloma virus, VIA, Visual Inspection with Acetic acid, WHO, World Health Organization

## Abstract

Our objective was to assess the health facility related factors that cause delays in cervical cancer diagnosis at a primary healthcare level in Rwanda. Healthcare providers in outpatient clinics at 10 health centers in Kigali city and the Eastern province of Rwanda were surveyed. Eighty-five healthcare providers participated; 83.5% were nurses and the remainder were midwives. Only 15 (17.6%) reported prior training on visual inspection with acetic acid (VIA) cervical cancer screening, and they were distributed among 6 of the 10 health centers surveyed. However, 76.5% of respondents reported that at least one person was trained in VIA at their health center. The basic equipment necessary for cervical cancer evaluation was reported to be generally available. Overall, only 31.8% of participants had good basic knowledge level on cervical cancer screening. No association was found between respondents’ knowledge about cervical cancer screening and profession, education level, work experience or reported prior training on VIA. There is a gap in the number of primary healthcare providers with the skills to perform pelvic exam and VIA cervical cancer screening at health centers in Rwanda. As health centers are the first point of contact for patients with the healthcare system, there is a need to improve their knowledge and skills in performing cervical cancer screening and detection.

## Highlights

• Cervical cancer is the fourth most common cancer in women despite being a preventable disease.
• Both patient and health system factors contribute to delay in diagnosis.
• Lack of knowledge and facilities create a gap in cervical cancer screening and prevention services.
• Coordinated effort is needed to integrate a functional cervical cancer prevention program into the Rwandan health system.

## 1 Introduction

Cervical cancer is the fourth most common cancer in women worldwide (Sung et al., 2021). In 2020, an estimated 342,000 deaths worldwide were due to cervical cancer (Sung et al., 2021). About 90% of deaths due to cervical cancer occur in low and middle-income countries, and if current trends continue it is projected that there will be a nearly 30% and 50% increase in cervical cancer deaths by 2030 and 2040, respectively (Ferlay et al., 2018, Sung et al., 2021). Low- and middle-income countries, especially where the disease burden is already the highest, including Eastern Africa, will be most affected (Ferlay et al., 2018, Sung et al., 2021). Cervical cancer is a preventable disease due to its long pre-invasive phase, thus providing opportunities for screening and early detection (Bradford and Goodman, 2013).

Improving cervical cancer control in high disease burdened countries is dependent on expanding cervical cancer screening and prevention strategies to all at-risk women.

In Rwanda, cervical cancer ranks as the leading cause of female cancer deaths, with an estimated 1,229 newly diagnosed cases and 829 associated deaths in 2020 (IARC, 2021). The high mortality rate associated with cervical cancer in Rwanda is in large part due to delays in diagnosis. Currently, Rwanda has no national cervical cancer screening program. A study performed at Butaro Cancer Center in Rwanda showed that 97% of cervical cancer patients enrolled from July 2012 to June 2015 had stage II disease or above (Davey et al., 2017). Delay in diagnosis is a major concern in cervical cancer control, with both patients and healthcare providers contributing to these delays (Gyenwali et al., 2014, Zeleke et al., 2021, Behnamfar and Azadehrah, 2015). It is estimated that in low income countries, only 5% of women are screened for cervical cancer (Bradford and Goodman, 2013).

In 2013, cervical cancer screening with VIA was introduced in Rwanda in a few district hospitals and health centers but was limited in uptake and not integrated into the public health system due to the lack of dedicated screening clinics (Binagwaho et al., 2013). Cervical cancer screening is offered in some public and private health facilities using Pap smear and VIA. The type of screening offered is determined by the staff and resources available at individual health facilities. Screening is opportunistic and typically provided per patient request. As there are no clinics dedicated solely to cervical cancer screening, most patients diagnosed with cervical cancer are evaluated because they are symptomatic, accounting for the large percentage of cervical cancer patients diagnosed at an advanced stage (Davey et al., 2017).

Primary healthcare in Rwanda is composed of health posts and health centers where nurses and midwives are the primary providers (MOH Rwanda, 2017). The community health centers are often supported by ancillary services including laboratory, social work, environmental health officers and clinical officers (MOH Rwanda, 2017, RAHPC, 2018). Patients first consult the primary healthcare facilities and are referred to secondary and eventually tertiary facilities as per the healthcare provider’s assessment (MOH Rwanda, 2017). There are no dedicated cervical cancer screening clinics operating at the primary health care level and information about the ability of the primary health care facilities to carry out cervical cancer screening services is not available. Multiple patient and health system factors can contribute to delays in diagnosis of cervical cancer which ultimately contribute to reducing cancer survival rates. (Gyenwali et al., 2014). The aim of this study was to assess the challenges encountered by healthcare providers in performing cervical cancer screening at the primary health facilities in Rwanda that contribute to delays in cervical cancer diagnosis.

## 2 Methods

This was a descriptive, cross-sectional study evaluating healthcare providers in outpatient clinics at health centers (HC) in Rwanda. Ten of the 508 HC in the country were selected to include both urban and rural areas; 7 of them are located within Kigali city and 3 located in the Eastern Province. No major differences are known to exist in HC in different provinces due to the relatively small size of the country and standardized services throughout. An average of 11 staff per HC rotate in the outpatient clinics. HC from Kigali City included Kabuga, Masaka, Busanza and Nyarugunga in Kicukiro district and Muhima, Gitega and Biryogo, located in Nyarugenge district. The HC from the Eastern Province included Muyumbu, Nyagasambu and Musha in Rwamagana district. The sample size was calculated by using surveysystem.com to determine how many surveys would need to be performed to obtain adequate sampling of the target population (Creative, 2012). The total population of health care workers in the 10 clinics was 110 and a confidence level of 95% and a margin of error of 5% were chosen which generated a sample size of 86 respondents. All healthcare providers involved in outpatient clinics at the HC selected were eligible for the study and participants were enrolled after signing an informed consent. The survey was conducted from December 2018 through January 2019.

Data was collected on a hard copy structured questionnaire, adapted from previously published studies to the local context (Khanna et al., 2019, Heena et al., 2019, Kress et al., 2015, Cham, 2018). It was translated into Kinyarwanda and back translated into English. The questionnaire consisted of 3 sections including participant demographics, basic cervical cancer screening knowledge and availability of facilities and supplies. The demographics section recorded the respondent’s profession, education level, work experience and training on VIA cervical cancer screening. VIA training in Rwanda typically consists of workshops or short courses and/or on-site training. Knowledge assessment section consisted of 6 questions with 11 possible Yes/No responses. A score of 1 or 0 was attributed to the answer of Yes or No, respectively. The maximum expected score was 11 and minimum score was 0. After aggregating knowledge scores, we used the original Bloom's cutoff points to categorize knowledge levels, where participants with a score of 80–100% were categorized as good knowledge, 60–79% moderate knowledge and below 60% categorized as poor knowledge (Khanna et al., 2019). The facilities and supplies section included questions on availability of cervical cancer screening services, staff trained on VIA screening and basic equipment for cervical cancer evaluation at the study HC.

Electronic data entry was performed using Microsoft Excel worksheet. After data cleaning, data was imported into IBM SPSS statistics for Windows software version 20 for analysis. Descriptive statistics were used to summarize demographic characteristics of respondents, distribution pattern of healthcare providers and availability of facilities and supplies for cervical cancer screening. Chi-square test was used to test the association between good knowledge level and participant demographics with significance level set at 0.05.

This study received approval from the University of Rwanda College of Medicine and Health Sciences Institutional Review Board (No 083/CMHS IRB/2018). Authorization for data collection was obtained from respective ethics committees of Muhima, Masaka and Rwamagana District Hospitals whose catchment areas include the 10 HC selected for this study.

## 3 Results

In total, 85 of 86 healthcare providers completed and returned the questionnaires, for a 98.8% response rate. Demographics of respondents are shown in Table 1. The level of education attained by participants was defined as follows: an A0 education is equivalent to a Bachelor’s degree, an A1 education level is an advanced diploma and an A2 education level, also called enrolled nurses, is a high school degree offered in the past but no longer exists on the Rwandan academic curriculum as of 2007 (Mukamana et al., 2015).

**Table 1:** Demographics of respondents (n = 85).

		Frequency	%
Profession	Nurse	71	83.5
	Midwife	14	16.5

Education level	A2	30	35.3
	A1	51	60.0
	A0	4	4.7

Work experience	0–2 years	16	18.8
	3–5 years	18	21.2
	6–10 years	17	20.0
	greater than 10 years	34	40.0

Trained in VIA cervical cancer screening	No	70	82.4
	Yes	15	17.6

A0 = Bachelor, A1 = Advanced diploma, A2 = Enrolled nurse.

**Fig. 1:**
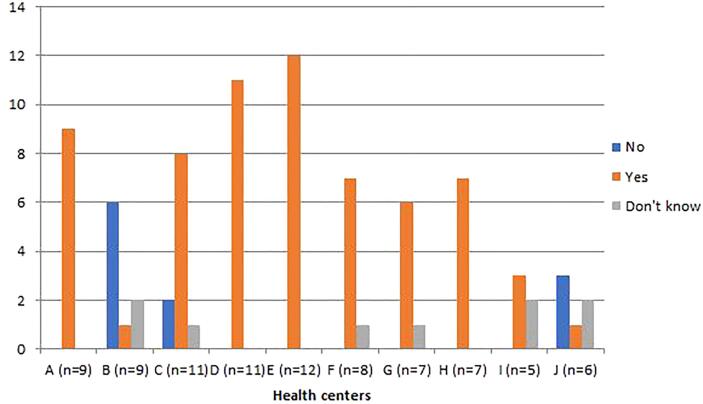
Availability of staff trained on VIA cervical cancer screening (N = 85).

**Fig. 2:**
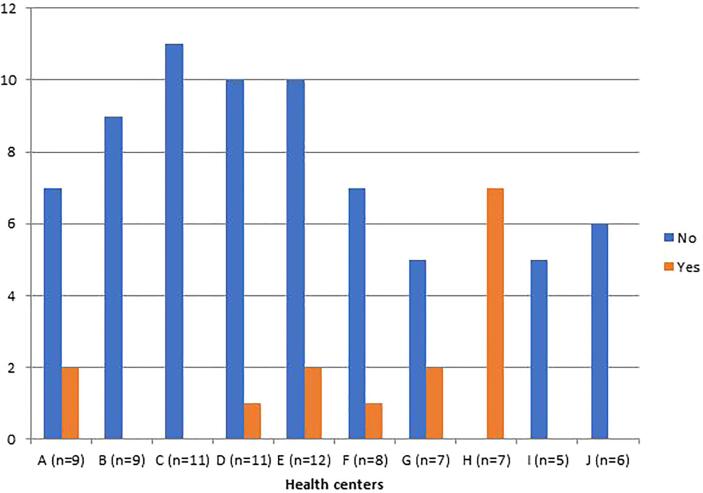
Staff personally trained on VIA cervical cancer screening (N = 85).

Data regarding the distribution of healthcare providers trained on VIA cervical cancer screening are shown in Fig. 1, Fig. 2. Of the 85 respondents, we found that only 15 (17.6%) reported having received training on VIA and were employed in 6 out of the 10 HC sites included in this study. However, at all HC at least one respondent reported the presence of one or more staff trained in VIA cervical cancer screening at their respective HC. Even at centers where a worker confirmed having trained in VIA, other respondents at the same center reported there was no one trained in VIA or they did not know if anyone was trained. This suggests there is uncertain knowledge of VIA services that are available at some HC by the healthcare workers themselves. In addition, 12/85(14.1%) and 33/85 (38.8%) of providers reported lack of training and lack of experience at performing a pelvic exam as the primary reason a pelvic exam was not performed even when indicated by the patients’ presenting symptoms.

**Fig. 3:**
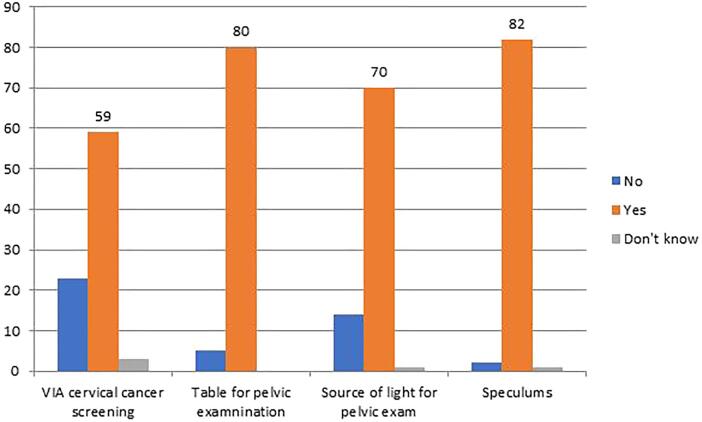
Availability of facilities (N = 85).

Barriers in the healthcare system that were reported include the availability of facilities and supplies for cervical cancer screening at HC as summarized in Fig. 3. Fifty-nine of 85 (69.4%) respondents in our study reported that cervical cancer screening is available at their HC and that the basic equipment for screening services, including examination table, light source and speculums, were generally available. However, when asked what the primary reason was for not performing a pelvic exam when indicated for symptoms of suspected cervical cancer, 16/85 (18.8%) responded that it was due to lack of appropriate materials. Not surprisingly, lack of adequate time, due to the excessive number of patients waiting for their services, was reported as the primary reason by 23/85 (27.0%) of providers.

In evaluating the basic cervical cancer screening knowledge of the healthcare workers, 27/85(31.8%) of participants were found to have good knowledge, 43/85(50.6%) moderate knowledge and 15/85(17.6%) poor knowledge. The results of bivariate analysis on the association between the respondents’ good knowledge level and different characteristics are shown in Table 2. We found no significant association between knowledge level and respondent profession, education level, work experience or reported past training on VIA screening (p = 0.329, 0.941, 0.187 and 0.281, respectively).

**Table 2:** Bivariate analysis: Respondents' characteristics and good knowledge level.

Good knowledge level		No n (%)	Yes n (%)	P value
**Total (N = 85)**		**58 (68.2%)**	**27 (31.8%)**	–

Profession	Nurse (n = 71)	50(70.4%)	21 (29.6%)	0.329
	Midwife (n = 14)	8 (57.1%)	6 (42.9%)	

Education level	A2 (n = 30)	20 (66.7%)	10 (33.3%)	0.941
	A1(n = 51)	35 (68.6%)	16 (31.4%)	
	A0 (n = 4)	3 (75.0%)	1 (25.0%)	

Work experience	0–2 years (n = 16)	13 (81.2%)	3 (18.8%)	0.187
	3–5 years (n = 18)	10 (55.6%)	8 (44.4%)	
	6–10 years (n = 17)	14 (82.4%)	3 (17.6%)	
	>10 years (n = 34)	21 (61.8%)	13 (38.2%)	

VIA training	No (n = 70)	46 (65.7%)	24 (34.3%)	0.281
	Yes (n = 15)	12 (80.0%)	3 (20.0%)	

A0 = Bachelor, A1 = Advanced diploma, A2 = Enrolled nurse.

## 4 Discussion

Inadequate infrastructure and insufficient basic equipment and supplies are reported to be among the challenges to cervical cancer screening in resource limited settings (Rosser et al., 2015, Maseko et al., 2015). In Rwanda, the primary health center is the first point of access to health care and screening services for patients. These centers are also the entry point for referral to specialized services in a hierarchical system. Our study reported that cervical cancer screening is available at the majority of HC, however, lack of appropriate materials, adequate skills and time are all barriers to performing cervical cancer screening including a basic pelvic exam. Our results are supported by a study performed at a tertiary level facility in Kigali which found that 33% of symptomatic cervical cancer patients did not have a speculum exam at the referring lower level facilities prior to referral despite the clinical suspicion of cervical cancer (Ruzigana et al., 2017). These findings are concerning, considering that a basic speculum and/or pelvic exam is the primary evaluation necessary to perform screening or evaluate a symptomatic cervical lesion.

The present study found that only 31.8% of participants had a good level of knowledge on cervical cancer screening. Provider knowledge on cervical cancer screening and prevention is often associated with professional background, education level, work experience or prior training in cervical cancer screening. However, our study failed to find an association between these variables and knowledge level. This could be attributed to an overall lack of training of healthcare workers on cervical cancer screening or the lack of routine practice on screening for the small number of providers who had received training in the past. Similar to other countries with limited resources such as Nigeria (Ifemelumma et al., 2019), all of the health care providers in this study agreed that cervical cancer is a public health problem in Rwanda. However, their knowledge, skills and confidence level on offering cervical cancer prevention and detection services is lacking.

This study also demonstrates the scarcity of staff trained in VIA-based cervical cancer screening at HC in Rwanda. HPV DNA testing is recommended by the WHO as the method of choice for cervical cancer screening when possible. However, in resource limited settings, VIA is the method considered by WHO to be the most feasible and cost-effective (Bradford and Goodman, 2013, Albert et al., 2021, WHO, 2013). We found only 17.6% of respondents reported having received training on VIA screening. These findings are similar to reports in other limited-resource countries where there are shortages of staff, specifically those trained in cervical cancer screening. A survey done in Kenya evaluating providers’ perceptions on barriers to cervical cancer screening found that 62% reported inadequate staffing as the primary barrier to cervical cancer screening and 60% reported insufficient training of staff as the primary barrier (Rosser et al., 2015). Similarly, findings in Malawi showed lack of readily available staff to offer cervical cancer screening and prevention services (Maseko, et al., 2015). Despite initiatives to increase access to cervical cancer screening globally, there continues to be a need to expand VIA training programs.

In Rwanda, community healthcare workers have contributed significantly to decreased maternal morbidity and mortality through increased adherence to prenatal care follow up and increased health facility deliveries (Perry et al., 2017). These community healthcare workers could be of importance in increasing the uptake of cervical cancer screening services through community sensitization and follow up. It is important to emphasize, however, that beyond increased community awareness, some of the most basic skills such as ability to perform an adequate pelvic and speculum exam need to be taught to healthcare workers, as well as VIA. Since 2013, when the initial countrywide training for VIA cervical cancer screening in Rwanda occurred, the number of gynecologists has increased from 26 to 74 specialists, reported in 2018 (Small et al., 2019). This increased number of specialists has provided gynecologic care to more district hospitals throughout the country. With this increase, improved knowledge of cervical cancer screening, symptoms and evaluation should occur through knowledge transfer from specialists to healthcare providers at surrounding HC throughout the country. Along with this increase in training and awareness, dedicated cervical cancer screening clinics should be organized to allow adequate time and evaluation of women for screening or symptoms to improve early diagnosis of cervical cancer.

Our study was limited in its design as it was based on participants’ responses and did not assess the type, quality and quantity of cervical cancer screening service facilities and supplies available at the HC. In addition, we did not undertake an audit of the available resources and supplies at HC to correlate with participants’ responses. For further evaluation of the actual needs in the system, we believe important next steps would be to physically assess the quality and quantity of equipment available for cervical cancer screening programs and identify barriers to cervical cancer screening completion at HC with available staff trained in VIA screening. This would then allow us to explore strategies for integrating a cervical cancer screening program into the current system.

In 2020 the WHO launched the Global Strategy to Accelerate the Elimination of Cervical Cancer as a Public Health Problem, with the goal of reaching an age-standardized incidence rate of less than 4 per 100,000 women. To achieve this goal globally within this century, initial targets for the first decade were outlined for the three pillars of action: to provide HPV vaccination to more than 90% of girls, to screen more than 70% of eligible women, and to ensure that 90% of women with cervical pre-cancer and invasive disease are able to access treatment and palliative care (WHO, 2020). Rwanda has been a global leader in HPV vaccination, with vaccination rates of girls over 90% since 2011 (Sayinzoga et al., 2020). A coordinated effort is needed to integrate a functional cervical cancer screening program into the Rwandan health system. Improving knowledge about cervical cancer screening and expanding access are key elements to improving cervical cancer control in Rwanda.

## CRediT authorship contribution statement

**Charles Nkurunziza:** Conceptualization, Methodology, Writing - original draft. **Rahel Ghebre:** Writing - review & editing. **Urania Magriples:** Writing - review & editing. **Diomede Ntasumbumuyange:** Writing - review & editing, Supervision. **Lisa Bazzett-Matabele:** Conceptualization, Methodology, Writing - review & editing.

## Declaration of Competing Interest

The authors declare that they have no known competing financial interests or personal relationships that could have appeared to influence the work reported in this paper.
